# Treatment of Malignant Diseases

**Published:** 1891-02-14

**Authors:** 


### SURGERY.

### TREATMENT OF MALIGNANT DISEASES.

At the present time, when so much labour is being devoted to the investigation of the ætiology of cancerous diseases, it behoves us to be careful not to lose sight of the graver question of treatment. The first point has been worked out by numerous observers from various aspects. M. Alexandre has investigated the blood in cases of malignant diseases of different varieties and in various organs, and finds that, with the exception of epitheliomata, the white corpuscles are more or less markedly increased in them; and he has noted six cases in which examination after removal of the neoplasm has shown a subsequent diminution of this excess. Scheurlein, Schill, Neisser, Darier, Albarran, Thoma, and others, and, quite recently, Russell of Edinburgh, have described various forms of micro-organism in the carcinomata or sarcomata which they incline to regard as the root of the evil, whereas Ballance and Shattock have obtained only negative results in their search for an organism. But whatever may ultimately be discovered to be the true cause of cancer, the important question for our patients and ourselves is, How are we to eradicate it when it is there? Our hopes of effecting a cure will depend largely on what view we accept as to the nature of the disease, and its methods of spreading and generalising. He who believes in a primarily local neoplasm is bound, if he be consistent, to act up to this belief in his treatment of the disease. The question as to whether malignant new growths are or are not in the first place strictly local abnormalities of cell growth is not, and probably will not for some time, be settled; but using what evidence we have, it is incumbent on us to come to some understanding with ourselves as to which hypothesis we are going to accept for working purposes. The doctrine of local origin has certainly the preponderance of support, and the main arguments in its favour may be summed up as follows: (i.) Its commencement as a single tumour; (ii.) the normal health enjoyed by patients during its primary single stage; (iii.) its method of spreading, and the inevitable identity of the primary and secondary growths; and (iv.) the undoubted fact that cures have been effected by removing the primary growth. The main objections are: (i.) Recurrence after apparently complete removal of the primary growth; (ii.) heredity; and (iii.) its varying degree of virulence and rapidity of growth in different cases. These, when examined, will not be found to have much weight. As to the first objection, it will be clear when we come to discuss the methods of recurrence of these growths, that what is thought to be a complete removal of a primary cancer may be so only in appearance. Heredity, undoubted though it is in a certain percentage of cases, is equally active in tumours of a perfectly benign nature, and is not to be confounded with constitutionalism. Lastly, degrees of virulence depend much more upon the particular variety of malignant growth present than on the individual attacked, and the same may be said of rapidity of growth with the additional factor of vascularity; for example, a carcinoma appearing in an active and in a quiescent breast will grow with very different degrees of rapidity in the two cases, the rate of increase in the former, owing to the increased blood supply attending its functional state, far surpassing that in the latter. In this the cancerous neoplasm is only obeying the law of all growing tissue.

Now a word as to the method of extension of malignant growths. I am not now speaking of the well-known routes of secondary dissemination by lymphatics or blood-vessels, but refer to the local contagion around the primary nodule which precedes it. This consists of a small-celled proliferation around the obviously malignant growth, which shades off gradually into healthy tissues, and which tends to spread furthest along the lines of blood vessels and lymphatics. This "latent zone of infection" is not appreciable to the eye or to touch, and yet doubtless in most cases (if not in all) contains already in some part the essential malignant element. Even in encapsuled sarcomata Cross has pointed out that this "latent zone" may exist before any definite eruption of the sarcomatous tissue has taken place through the capsule walls. When this does take place it spreads rapidly along the lines of the vessels. Admitting this to be the usual state of a primary malignant nodule, the reason of the lamentable frequency of recurrence after apparent extirpation is not far to seek.

Heidenhain has lately made some important observations in this connection in the case of mammary cancer. In eighteen such cases the gland was carefully examined microscopically. In twelve cancerous elements were found at the base of the gland, and in the remaining six the base was healthy. He accordingly gave a favourable prognosis in these last, but an unfavourable one in the first twelve cases. His predictions were confirmed by the results. From these cases he formulates the following conclusions: 1. The thin pectoral fascia is closely connected with its muscle, and cannot be cleanly separated from it. 2. In thin women the gland is distinct from this fascia, but in fat subjects parts of the gland are adherent to it and the muscle. 3. If the gland contains even a small nodule of cancer the breast may be considered to be diseased throughout. The epithelial cells of the acini and the connective tissue become infected, and the lymphatics carry these to a distance. One possible cause of recurrence may therefore be acini left behind in outlying parts. 4. These cancerous elements stretch with the blood vessels and lymphatics from the gland into the retro-mammary fat. The disease may also spread to the connective tissue around secondary lymphatic glands. 5. If the gland is unattached to the Pectoralis major this muscle is usually healthy, but if any part of it is adherent to it, it is infected, and infected in its entire extent. Heidenhain urges, therefore, that when the tumour is free of the Pectoralis it should be removed with the superficial part of the muscle, but when adherent to it the whole muscle should be removed in its entirety.

These observations will serve to indicate the kind of operative treatment we desire to urge. It cannot be too strongly insisted that at present no method of treatment is of any curative value except free removal of the morbid growth at the beginning. Of the treatment by Chian turpentine, or oyster shells, we intend to say nothing, as they have both failed, so far, to establish their claims. Destruction of cancerous growths by escharotics or scraping—except as possible palliatives in advanced cases—is condemned by modern surgery. There is, however, one other treatment of cancerous growths advocated by Inglis Parsons, which seems well worthy of a trial in cases which have spread beyond the reach of the knife. We refer to the passage of a powerful interrupted voltaic current through the tumour, and the surrounding tissues for some inches. This is carried out under an anæsthetic, and the intensity of the current used is increased progressively from 10 to 600 milliampères. It requires to be used 50 to 100 times. Mr. Parsons reports four cases in which the growth of the tumour was arrested by this method.

Having so far cleared the ground in favour of operative treatment in these cases, a word or two may be said as to the way in which these operations should be carried out: (1) If the new growth is just under the skin, even if this is not involved, a large piece of the skin over it must be removed; and if the skin is already implicated, the amount excised must be far in excess of what naked eye appearances would seem to suggest, and especially must this be so in the line of lymphatics or vessels leaving the growth, as, for example, towards the axilla in carcinoma of the breast. The same rule of very wide removal applies to the connective tissue in which the growth is embedded. (2) In carcinomata the nearest lymphatic glands should be always excised—with their surrounding connective tissues (a very important point), even if they are not sensibly enlarged—wherever this practice is possible. Küster, Kocher, and Mitchell Banks have directed special attention to this point, and in this way the latter has obtained, in cases of mammary cancer, a degree of immunity from recurrence far greater than that usually seen. (3) In the case of malignant disease of bones, the very freest removal is necessary, if success is to be obtained. It is a golden rule to remove the entire bone affected—with a very doubtful exception in favour of myeloid sarcoma in the articular end of a bone — and this means amputation at or above the next joint, even if it be the hip-joint. But in some cases—especially if seen late—the muscles inserted into the bone become contaminated, and the malignant infection will then spread very rapidly throughout their extent, so that in such cases, as Erichsen recommends, the amputation should extend, wherever possible, above the origins of the muscles inserted in the neighbourhood of the disease. It is pretty generally recognised that the earlier a malignant tumour is removed the better; but how often does the surgeon's courage fail when the question of freedom of removal comes practically before him. It is surely not too much to say that no resulting deformity is of the slightest consequence compared with the successful eradication of the local stage of the disease. No provision for the better adaptation of the edges of the wound, and no shrinking (however natural) from a dangerous (if practicable) operative measure can justify any default in this direction. If such a free removal of malignant diseases were more generally adopted, I believe that amongst those cases which come for treatment at a reasonably early stage, our table of recurrence would show a marked decrease, and many valuable lives would be spared to carry on their work, and no small increase of honour accrue to the practice of surgery. And yet even if this happy result is not obtained in any case, as Shrady points out, a well-devised operation will probably prolong life, and nearly certainly diminish the total suffering.

